# Spatial Concept Learning: A Spiking Neural Network Implementation in Virtual and Physical Robots

**DOI:** 10.1155/2019/8361369

**Published:** 2019-04-01

**Authors:** André Cyr, Frédéric Thériault

**Affiliations:** ^1^School of Psychology, University of Ottawa, Ottawa, Ontario, Canada; ^2^Department of Computer Science, Cégep du Vieux Montréal, Montréal, Quebec, Canada

## Abstract

This paper proposes an artificial spiking neural network (SNN) sustaining the cognitive abstract process of spatial concept learning, embedded in virtual and real robots. Based on an operant conditioning procedure, the robots learn the relationship of horizontal/vertical and left/right visual stimuli, regardless of their specific pattern composition or their location on the images. Tests with novel patterns and locations were successfully completed after the acquisition learning phase. Results show that the SNN can adapt its behavior in real time when the rewarding rule changes.

## 1. Introduction

Mastering abstract concepts seems like a key to reach a higher level of cognition, allowing animals to gather more complex knowledge [1]. Concepts save time by avoiding learning every stimuli, regrouping them in general categories to deal with new situations. According to Zentall et al. [2], three main hierarchical types of abstract concepts are defined. Perceptual or natural abstract concepts consist in finding physical similarities between different objects or stimuli and are a first type of categorization. A second type, relational concepts, concerns the general rule or abstract relationship between stimuli that is not directly related to their specific physical attributes. In that sense, it is a second-order process [3, 4]. For example, sizes (such as small and large) are abstract categories that are determined by comparing the sizes of the presented objects. Thus, the dimensional relationship is not tied to the exact physical size of the objects but compared and developed from experience. Finally, associative or functional concepts imply that one stimulus or characteristic is interchangeable with another one (i.e., Dogs-Barking). This paper focuses on spatial abstract concepts as a prior step toward achieving a relational above/below neural circuit.

There is an abundant collection of empirical data on relational concepts, as well as in the literature. Animal models and methodologies are also numerous with many levels of comparison [5–9]. Recently, this higher cognitive process was explored with invertebrates. Astonishingly, it was shown that bees could learn several different types of relational concepts, despite having a small brain that consists of less than one million neurons [10–15]. Even if some progress is made to relate the learning process and the neural substrates [16], no precise neural circuit is currently known to explain concept learning from a complete sensory to motor architecture, be it natural or artificial. Also, the relationship between perceptual and relational concept levels remains mostly unexplored from a computational neurorobotic perspective.

Neural modeling is one computational tool that that maybe helpful for approaching this problem, more precisely by elaborating a precise artificial neural circuit that correlates the behavioral observations. Few articles have explored the abstract concept learning process phenomenon from this angle. Therefore, this article seeks to further study the topic under a spiking neural network (SNN) paradigm. Moreover, this research also goes a step beyond an SNN by implementing the whole cognitive process in a complete virtual and physical neurorobotic model [17]. This allows validation of the proposed computational model in a brain-body-environment or embodied cognitive context [18].

SNNs are bioinspired neural models that have emphasis on single spike events and their temporal-coincidental relations [19, 20]. Generally, the learning rule used from these neural models is based on synaptic changes from a spike-timing dependent plasticity (STDP) process [21–23]. As such, this paper uses a specific SNN model to sustain the representation of a spatial concept learning process.

In this study, a spatial visual task with different images composed of horizontal/vertical and left/right patterns are shown in front of a static robot. From an operant conditioning procedure, the robot has to decide which side to choose (left or right). Hence, from reinforcements, it learns to associate different spatial relations, independently of specific stimulus patterns shown and their locations. This visual learning scenario is partially inspired from the one made with bees [11, 24], which fully succeeded in learning two relational abstract concepts (above/below, left/right, and same/different) with generalization transfer tests. This paper is in the continuity of our previous work, which was to build an SNN that sustains the identity concept learning process in the neurorobotic domain [25].

The next section describes the methodology and the details on the learning protocol. It is followed by results, highlighting the spatial concept learning process from the synaptic to the behavioral changes. The last section contains a discussion on the current model's limitations and the future perspectives of this learning model.

## 2. Methodology

### 2.1. Protocol

**Figure 1:**
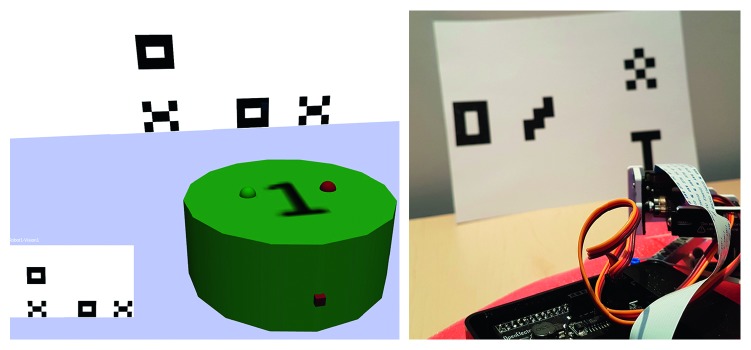
Similar virtual and physical environments, showing the robots and their view. On the left side, the virtual environment displays the robot's view on the bottom left part. In this case, it consists of a left vertical and a right horizontal image, composed of two different patterns (O and X).

**Figure 2:**
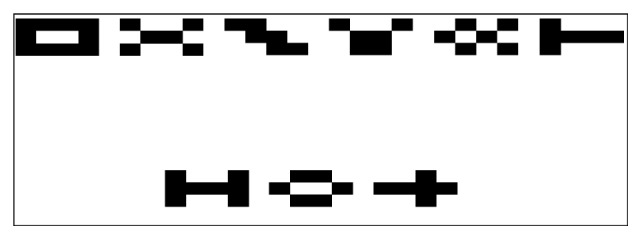
All patterns used in this study. The top six patterns are shown in the first part of the experiment. The three lower patterns represent the novel patterns used at the end of the simulation.

The visual task consists in learning horizontal/vertical and left/right spatial concepts. Images are projected in front of a robot. Each of them has two sides (left and right): one side contains two black/white motifs aligned vertically and the other side contains two motifs aligned horizontally (Figure 1). The first experiment consists of grouping patterns (horizontal or vertical), which are permuted on three possible positions on each side. Thus, all images are first randomized for the side of the horizontal and vertical patterns, second for the position, and third for the individual stimulus patterns composing them (Figure 2). Images cover the whole field of view of the robot's camera. The second experiment tests stimuli on novel locations, once the learning phase is completed. Finally, a third experiment allows to validate the SNN under less precise conditions, by using a real robot.

Following an image capture, the robot takes a dichotomous left or right decision according to a chosen stimulus, randomly selected prior to learning. This action is manifested by directly rotating its motor towards it. From a conditioning procedure, a reward is consistently applied on the vertical or the horizontal motif, depending on the desired learning rule. Along with the task and with few positive reinforcers, the robot learns the horizontal/vertical or left/right relation, ignoring the exact individual pattern features as well as its location on its side. To validate the robustness of the SNN, the experiment ends with the presentation of novel patterns at new locations.

### 2.2. Architecture

**Figure 3:**
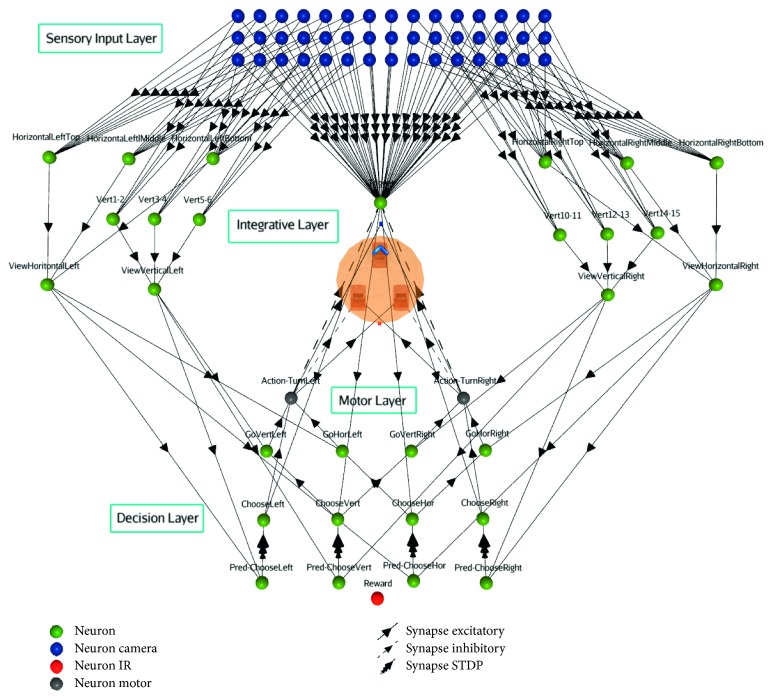
Full view of the SNN architecture, composed of the robot and four different functional neural layers.

The neural circuit is organized into four basic layers: a sensory input layer, an integrative layer, a decision layer, and a motor output layer (Figure 3). The sensory visual neurons are linked to a camera that captures images of 4 : 3 ratio. These neurons are arranged in a 3 × 15 array, with each of them overlapping a different spatial section, hence completely covering the visual field. In this experiment, sensory neurons only integrate black intensity with numerical values. These are averaged and normalized in a percentage scale. Therefore, the spiking activities of sensory visual neurons reflect the stimulus patterns shown in front of the robot. Once an image of the robot's view is captured, a cooldown prevents the camera from triggering before an action is made. Otherwise, constant stimulus inputs from this layer would prevent the SNN from integrating and acting on a single image.

The sensory input layer forwards signals to integrative neurons. These are topographically organized in a neighborhood configuration, separated in left/right and upper/mid/lower logical sections. In the current model, the first level of integration is composed of 12 neurons (six for vertical and six for horizontal detection). This allows the SNN to react to local stimuli. More precisely, each integrative element can respond to any vertical or horizontally displayed black stimulus. A second integrative level regroups all horizontal and vertical neurons for each side (ViewVerticalLeft, ViewVerticalRight, ViewHorizontalLeft, and ViewHorizontalRight).

From the integrative neurons, signals are propagated to the decision layer, more precisely to the Predictor neurons. Those Predictor neurons are linked to their associated Choose neuron (ChooseLeft, ChooseRight, ChooseVert, and ChooseHor) with a weak excitatory synapse and a synaptic learning rule (STDP) and are also connected to the action layer. Prior to learning, Predictor neurons cannot trigger Choose neurons alone. As rewards are given, the STDP rule strengthens those specific synapses. This eventually allows the correct Predictor to trigger its associated Choose neuron. Rewards are simulated by moving a block in front of an infrared sensor located at the back of the robot. In this study, the learning rule from STDP needs a third factor (the reward) to be activated [26, 27]. When no reward is given, it implies that the robot took a wrong decision and the synapse strongly weakens.

The decision layer also contains Go neurons (GoVertLeft, GoVertRight, GoHorLeft, GoHorRight). For example, when the horizontal Choose neuron spikes, the Go horizontal neuron allows the proper action (turn left/right) to be done, depending on where the horizontal stimulus is located.

The action layer consists of two motor neurons (Action-TurnLeft, Action-TurnRight), orienting the robot towards the chosen side. Prior to learning, when a pattern is detected in the sensory visual layer, a randomized action is triggered by sending a delayed signal to motor neurons. This action could eventually be bypassed from the Predictor neurons in the decision neural, after learning.

### 2.3. Neural Dynamic

The spiking neural model used in this paper and the neural architecture were achieved with the SIMCOG software [28]. The neural dynamic is based on standard properties, which are membrane potential variation (equations (1), (3), and (4)), nonlinear integration of excitatory/inhibitory inputs (equation (2)), threshold for spike events, absolute refractory period, and an after spike hyperpolarization state. Since the neural circuit is well defined, the tuning of the starting synaptic weights was manually adjusted prior to launching the final experiment (supplementary materials for starting synaptic weight values at http://aifuture.com/res/2018-spatial). The learning rule used in the proposed model integrates a STDP function (equation (5)) only available for synapses in the decision layer.

Leaky integrator neural dynamic:

(1) $$\begin{array}{c} v_{\text{m}}\left(k\right)=f\left(v_{\text{m}}\left(k-1\right)+\sum v_{i}\right), \end{array}$$

where *v*_*m*_(*k*) = membrane potential at cycle *k*, *v*_*i*_ = synaptic input as calculated in equation (2), and *f* = membrane potential curve as calculated in equation (3).

General function describing the postsynaptic potential curve:

(2) $$\begin{array}{c} v_{i}\left(t\right)=\left\{\begin{array}{cc} ae^{-t/\tau }, & \text{if }t\leq t\text{ Max,}\\ 0, & \text{if }t>t\text{ Max,} \end{array}\right. \end{array}$$

where *a* = maximum amplitude (set from 2 to 20), *τ* = tau (set to 7), *t* = time since spike (cycle), and *t* Max = maximum duration of a PSP (set from 1 to 10 cycles).

Membrane potential function:

(3) $$\begin{array}{c} f\left(v_{\text{m}}\right)=\left\{\begin{array}{cc} g\left(v_{\text{m}},0\right), & \text{if }v_{\text{m}}<v_{\text{m}}\text{ Rest,}\\ v_{\text{m}}\text{Rest}, & \text{else if }v_{\text{m}}=v_{\text{m}}\text{ Rest,}\\ g\left(v_{\text{m}},1\right), & \text{else if }v_{\text{m}}<v_{\text{m}}\text{ Threshold,}\\ 100, & \text{else,} \end{array}\right. \end{array}$$

where *v*_m_Rest = membrane potential rest value (set as 43) and *v*_m_Threshold = threshold value (set as 65).

Membrane potential output:

(4) gvm,d=mineach v in vec where v>vm,if d=0,maxeach v in vec where v<vm,if d=1,

where vec = [4, 11, 18, 23, 28, 32, 36, 42, 43, 44, 45, 47, 50, 53, 58, 65, 100], ascending phase to reach threshold = exp(0.8+0.3 *∗* *t*)+40 for each *t* from 0 to 8, ascending phase from post action potential to rest=log  10(0.9+0.2 *∗* *t*) *∗* 100 for each *t* from 1 to 7, and action potential = 100.

General STDP function.

(5) $$\begin{array}{c} \Delta w=a\;*\;b\;*\;e^{-abs\left(t_{\text{post}}-t_{\text{pre}}\right)/c}, \end{array}$$

where Δ*w* = synaptic weight change, *a* = multiplicator factor (set to 1.0), *b*=1 when *t*_post_ > *t*_pre_, −1 when *t*_post_ < *t*_pre_, *c* = time-constant (set to 100/3), STDP coefficients for Δ*w*: duration of the synaptic change = 1000 cycles, max. synaptic change in one paired spike = 25%, and max. global synaptic change = 100%.

### 2.4. Physical Environment

After tuning the SNN parameters and evaluating them in a virtual world, it was embedded in a physical environment using a Raspberry Pi 3 mounted with a 160 × 120 resolution camera and two servomotors (for pan/tilt camera rotation). The objective of this simulation was to verify the SNN's capability to learn with less precise variables (i.e., timing of events, camera detection, etc.). To embed the SNN in the Raspberry Pi robot, it only required a single modification. Since the robot does not contain infrared sensors, the reward was instead given by displaying a red sheet of paper in front of it. Hence, an additional reward visual neuron was linked to the camera, in order to perceive the red color.

## 3. Results

**Figure 4:**
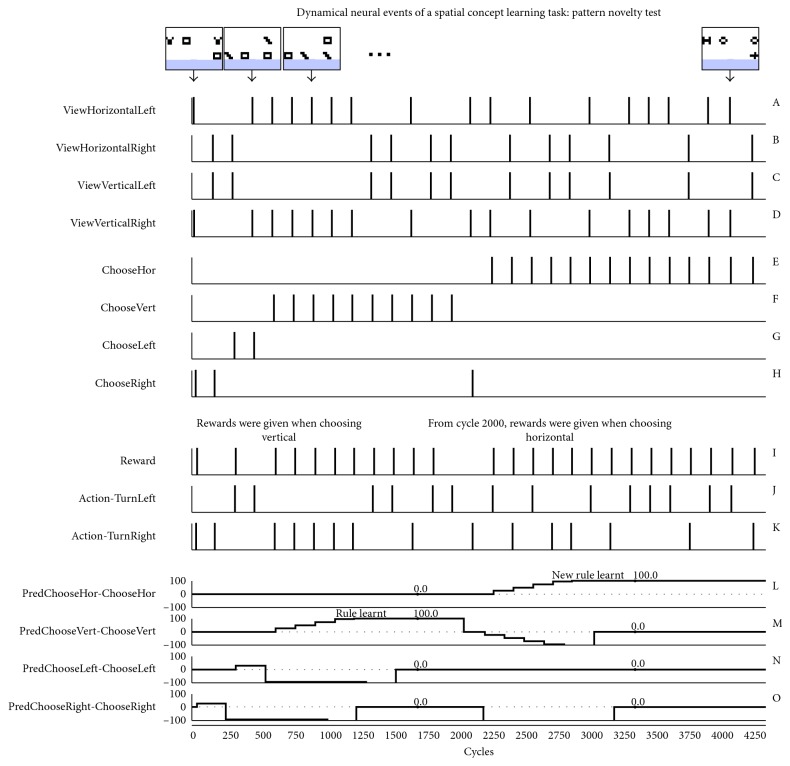
Dynamical neural representations of the virtual simulation, showing the SNN's behavioral adaptation according to given rewards. Graphics A–K represent neural spike events and L–O consist of synaptic STDP coefficients. The final test includes novel patterns composing the stimulus. In the top row of the figure, only few images from the whole set are shown for clarity, the arrow indicating the perceived stimulus.

Figure 4 represents the neural behavior dynamic of the main elements achieving the spatial concept learning task. For each trial, the sensory neural layer (3 × 15 array of neurons) captures the image with one horizontal pattern on one side and one vertical pattern on the other side. These are composed of two different black and white motifs (3 × 4 pixels). Three examples of the robot's view are shown at the top of the figure. The sensory layer forwards the signal to the integrative layer resulting in associated spike events of the four main neurons (graphics A to D). From these, a single Choose neuron from the decision neural layer (graphics E to H) emits an action potential.

In the first experiment, the desired output was set on the vertical stimulus. Then the rewarding rule was modified, as of cycle 2000, to give a reward when choosing the horizontal pattern. One can see that the SNN fully adapted its behavior even when changing the rewarding rules online. The reverse situation (learning horizontal stimulus before the vertical one) was also tested with no effect on the learning procedure (not shown). Since the image sequence is randomized, including both the patterns and the horizontal or vertical sides, several trials were done. In all cases, the SNN succeeded in learning, adapting its behavior according to the desired output.

At the beginning of the simulation, the synaptic links between the Predictor neurons and the Choose neurons are weak. Thus, the choice of action is random. During the experiment, a positive reinforcement (Graphic I) is applied when the SNN succeeds in choosing the correct action (Graphics J and K). This learning process is shown in graphics L to O with an increase in the synaptic weights from several rewards. The learning step factor was designed to reach the threshold point after three correct associations, but it could have been done differently for smooth learning or even to trigger a learnt response after a single correct trial. When the SNN constantly predicted the correct action, a last test was done with novel patterns (see example at cycle 4100).

**Figure 5:**
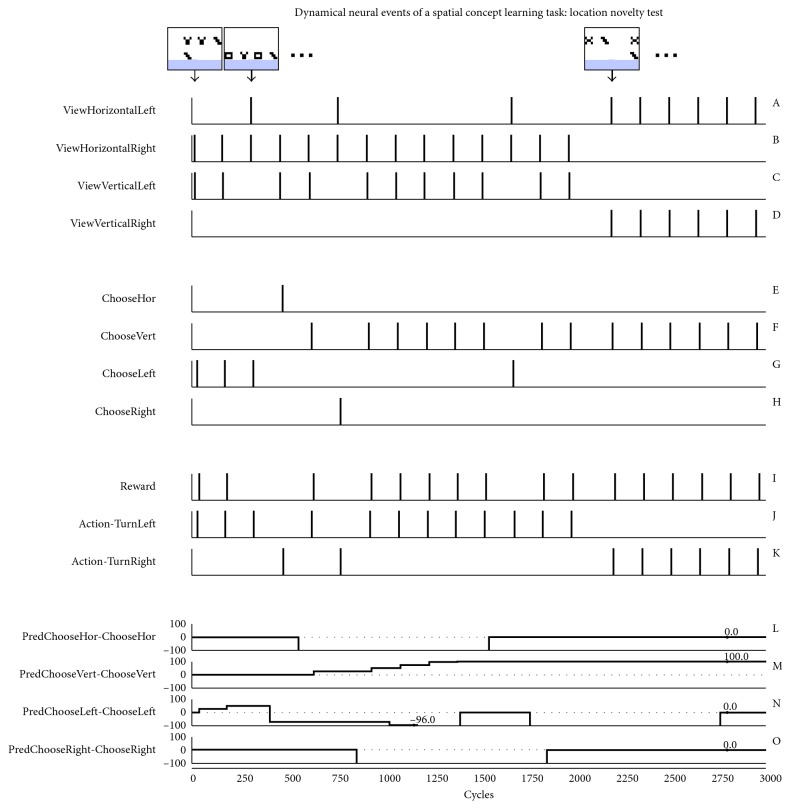
Dynamical neural representations of the virtual simulation, showing the ability of the SNN to adapt its behavior according to given rewards. Graphics A–K represent neural spike events and L–O consist of synaptic STDP coefficients. The final test includes a novel location of the stimulus. In the top row of the figure, only few images from the whole set are shown for clarity, the arrow indicating the perceived stimulus.

In the second experiment, most images were displaying verticals on the left side, up until cycle 2200 (Figure 5). This allowed the SNN to learn between two choices: left and vertical. To prevent the robot from only learning the left side rule (from the ChooseLeft neuron), few horizontal-left/horizontal-right images were shown (for example, see at cycle 250). After cycle 2200, vertical patterns were shown on the right, verifying that the SNN could still use the learnt vertical rule even though it was on a novel location.

The real experimentation, using the Raspberry Pi, gave similar results, though it was necessary to run the experiment a few times before succeeding. The main difficulty here was with respect to the timing and manual image adjustments in front of the camera; otherwise, it was not capturing images correctly in the sensory input layer. Also, since no infrared sensors were available on the real robot, the rewards were instead given by showing red colored papers in front of it, which were perceived by its camera. This added some artifacts during the simulation. Video and simulation results are available as supplementary materials: http://aifuture.com/res/2018-spatial.

## 4. Discussion

Abstract concept learning is thought to be a higher cognitive process and a key feature of intelligent natural species. The recent literature in neuroscience suggests that that even invertebrates with small brains could reach this level of complexity. This attractive fact stimulates the emulation of the cognitive phenomenon with a bioinspired artificial spiking neurons approach embedded in a neurorobotic model. One working hypothesis of this paradigm relies on a level of computational general intelligence level, based on functional cognitive processes that are related for specific physical body structures and environments. However, the simulation of a cognitive process from a precise artificial neural circuit and a given robot implementation does not intend to reflect a natural one, but only reproduce the function and the behavior with artificial substrates, grounded in a real-world context.

This project intends to be a prior step to reach the relational concept level, from designing a specific SNN associated to the spatial abstract concepts of horizontal/vertical and left/right. Beyond the main objective of simulating this learning process through a neurorobotic model, the present experimentation also serves as a prototype model to further study the development of a general neural design, which could sustain the three different types of concept learning, as well as several forms of concept inside each category.

In its current form, the SNN model is limited by its small visual scale (retina) and a single color perception (black). The SNN design is also restricted to detect perfect horizontal or vertical stimuli. Hence, it would be unable to perform when seeing an angled stimulus in front of it, which is another possible test for the generalization property. In the same perspective, scaling up or down stimuli was not possible in this experiment, again from the limitative capability of the retina. However, these issues could be corrected in future works. Furthermore, a higher discrimination would be a desirable feature to include in the present SNN model, since it is needed in the above/below and same/different relational concepts, as well as its full validation from transfer tests. However, we believe that the core neural layers of this architecture would remain and could be used in more related complex studies.

Does the relational concept learning process emerge from experiences and synaptic modifications of an existing neural circuit, or does it need the addition of new neurons as in the developmental neural phases? Is the relational concept structured in a bottom-up neural hierarchy? Does the first-order perceptual level of categorization sustain the second-order relational abstract concept? As a start toward answering those questions from a neurorobotic model perspective, the proposed SNN allows learning two spatial concepts from a specific set of neurons and synapses. At first, the learning rule was unknown for the robot, but as rewards were given, the SNN adapted its behavior from supervised reinforcements in an operant conditioning procedure. Also, the SNN exhibited a behavioral plasticity when changing the rewarding rule online.

In the present experiments, it is not necessary to discriminate stimulus patterns, for example, to differentiate the black square stimulus and the X shape stimulus. This lower level of perception was not required to achieve the spatial learning task for left/right and horizontal/vertical patterns. However, it certainly represents a critical step to reach the relational abstract learning level. For example, in the above/below scenario, determining the constant visual spatial referent while the location of the other visual pattern varies requires a perceptual discrimination and a functional action of comparison. This is a future work for our team to integrate the present model and build an SNN that links this spatial concept level to a second-order relational concept.

Another objective of this paper is to provide comparative experimental data between different computational robotic models, as well as developing benchmarks for testing incremental complexity scenarios in the field of abstract concept learning.

## 5. Conclusion

This paper shows that the proposed SNN, controlling virtual and physical robots, succeeded to learn the spatial concept of horizontal/vertical and left/right visual patterns from a conditioning procedure and synaptic modifications. This experiment intends to be a first step study to reach the second-order relational concepts as in the above/below case. We believe that this bioinspired approach may open new perspectives to reach higher artificial cognition in the neurorobotic domain.

## Data Availability

The complete access to all parameters and result data used to support this study, as well as the SIMCOG software, is available from the corresponding author upon request.
